# Medical specialists’ attitudes and practices towards childhood vaccination: a qualitative study in Armenia

**DOI:** 10.1186/s12887-022-03687-3

**Published:** 2022-10-29

**Authors:** Cath Jackson, Siff Malue Nielsen, Brigida Simonyan, Marine Kirakosyan, Marine Hovhannisyan, Gayane Sahakyan, Katrine Bach Habersaat

**Affiliations:** 1 World Health Organization Regional Office for Europe, Marmorvej 51, DK-2100 Copenhagen, OE Denmark; 2 National Center for Disease Control and Prevention, 49/4 Komitas Avenue, 375095, 37051 Yerevan, Armenia

**Keywords:** Vaccination, Immunization, Immunisation, Health worker, Armenia, Tailoring immunization programmes (TIP)

## Abstract

**Background:**

Childhood vaccination rates in Armenia are high. However, anecdotal evidence suggests that some health workers may advise against vaccination. The extent and reasons behind this are unknown. This study used the World Health Organization Tailoring Immunization Programmes approach to investigate medical specialists’ vaccination practices.

**Methods:**

Face-to-face interviews were conducted with 30 medical specialists (paediatricians, immunologists, neonatologists, neurologists, gynaecologists). Interviews explored their vaccination practices (recommending/administering), knowledge, attitudes and confidence. Data were analysed using the Framework approach and COM (Capability, Opportunity, Motivation) factors.

**Findings:**

Medical specialists were routinely consulted by parents about vaccination. They engaged in conversations, even if they did not administer vaccinations and lacked expertise. Vaccination recommendation was “selective”, influenced by their own vaccine hesitancy. Doctors administering vaccination used false contraindications to postpone vaccination. Multiple barriers and drivers to positive vaccination practices were evident, with differences between specialists administering/not administering vaccinations.

**Capability**

Drivers were knowledge of vaccination, vaccines, and vaccine-preventable diseases; with awareness and use of protocols for adverse events and contraindications (those with a vaccination role). Barriers were a lack of a detailed understanding of vaccination, vaccines, and vaccine-preventable diseases, especially amongst neonatologists and gynaecologists, and for HPV. Poor knowledge of adverse events and mixed knowledge of contraindications was evident, as was low confidence about conversations with parents declining vaccination.

**Opportunity**

Drivers were using “official“ guidance and professional information and feeling protected by the Government of Armenia should an adverse event occur. Conversely, barriers were a reliance on media/social media without considering credibility, peers not recommending vaccination, increasing parent demands and not feeling protected by the Government.

**Motivation**

Drivers were seeing vaccination as their responsibility (those who administer vaccinations); and generally supporting vaccination. Barriers were vaccine hesitancy, some anti-vaccination sentiments amongst neonatologists and gynaecologists and not seeing vaccination as their role (those who do not administer vaccinations).

**Conclusions:**

Applying a theory-informed approach allowed us to identify critical issues and possible solutions. High vaccination coverage may disguise underlying issues, e.g. false contraindications. We addressed gaps in the literature, with our geographical focus and study of medical specialists advising parents on vaccination, a widely used practice in this sub-region.

**Supplementary Information:**

The online version contains supplementary material available at 10.1186/s12887-022-03687-3.

## Background

Childhood vaccination rates in Armenia are consistently high. Data from the last 30 years [1] indicate coverage of close to, or above 95% for all childhood vaccines provided in the National Immunization Programme (NIP). In 2016 (the year prior to this study), full vaccination coverage was 92%, 94% and 97% (at 1 year, 2 years and 6 years of age respectively), achieving targets for national coverage stipulated in the European Vaccine Action Plan [2]. Consequently, the burden of vaccine-preventable diseases has been consistently low in Armenia. For example, no outbreaks were detected in 2016 [3]; and polio-free status has been sustained since 2002 [4, 5]. Childhood vaccination is mandatory in Armenia, with possible exemption/postponement based on contraindications.

Despite this positive vaccination context, challenges and threats to the success of the childhood vaccination programme have occurred along the way. Demand-side challenges include timeliness of vaccination (with less than 84% timely vaccination of first dose for most childhood vaccinations) [1] and increasing hesitancy and refusals particularly evident amongst urban parents [6, 7]. On the supply-side, inadequate knowledge amongst some health workers is an ongoing concern [7, 8], as are some negative attitudes to particular vaccines [9], increasing rates of false contraindications [8], and health worker concerns about being blamed for Adverse Events Following Immunization (AEFI) [7, 10]. Perhaps the most significant threat to the childhood vaccination programme is related to the introduction of the Human Papillomavirus (HPV) vaccine in December 2017 which was met with confusion, fear and resistance amongst health workers, parents and the media [11]. This was undoubtedly amplified by a highly effective anti-vaccination campaign that occurred a few months before vaccine introduction. In 2018, national coverage of the first dose was just 6% [12], prompting concerns amongst key stakeholders that this experience might have implications for the wider vaccination programme.

In 2017, with these challenges in mind, the Armenian National Centre for Disease Control and Prevention (NCDCP) decided to initiate a World Health Organization (WHO) Tailoring Immunization Programmes (TIP) project [13, 14]. TIP uses social sciences, ethnographic research techniques and behavioural insights methodology to support countries to diagnose barriers and drivers to positive vaccination behaviours amongst susceptible populations; to then design tailored interventions to increase vaccination coverage. The theoretical framework underpinning the TIP approach is the Capability-Opportunity-Motivation-Behaviour (COM-B) model [15] which identifies the inter-linked individual (capability, motivation) and context (opportunity) determinants of vaccination behaviours.

Following a situational analysis of the childhood vaccination programme and input from a stakeholder workshop, the decision was taken to focus the Armenia TIP project on medical specialists. These are doctors who are asked by parents for their advice about childhood vaccination, irrespective of whether they have a remit for administering these vaccines. Examples of these doctors are presented in Table 1. It is a widely used practice in Armenia (as well as in other countries in this geographical region) to refer parents with questions regarding vaccination to specialists, or for parents to independently seek advice from specialists regarding vaccination of their child, including neurologists, paediatricians and immunologists. The understanding amongst stakeholders was that there were medical specialists in Armenia who were recommending *against* vaccination. The extent of this practice and the reasons behind it were unknown. Globally, the importance of health workers’ recommendations for parents making childhood vaccination decisions is well documented [16–18]; however, the evidence relates predominantly to those with a remit for vaccination namely paediatricians, general practitioners, nurses and community health workers. Where studies have included medical specialists, they tend to comprise a small part of the sample and their views are not distinguishable from those of primary care health workers [10, 19]. There are some data on gynaecologists’ perspectives on their role in HPV [20] and influenza vaccinations [21].

**Table 1:** Summary of Participant Characteristics

	Yerevan		Tavush		Armavir		Total	
**Profession**	Hospital	Polyclinic	Hospital	Polyclinic	Hospital	Polyclinic	Hospital	Polyclinic
Paediatrician	4	6*	0	0	0	0	**4**	6*
Immunologist	1	1*	0	1*	0	0	**1**	2*
Neurologist	1	3	1	0	0	0	**2**	**3**
Neonatologist	4*	0	0	0	2*	0	6*	**0**
Gynaecologist	5	1	0	0	1	0	**6**	**1**
**Total**	**15**	**11**	**1**	**1**	**3**	**0**	**19**	**12**

*Note.* *Doctors who administer childhood vaccinations

In this paper, we present a qualitative interview study with medical specialists in Armenia. The aims were to:

1. Investigate medical specialists’ childhood vaccination practices, and their perceived barriers and drivers to positive practices.
2. Examine whether, and how, these responses vary for medical specialists who administer childhood vaccinations and those who do not administer childhood vaccinations.

## Methods

This was a qualitative, interview study conducted from September 2017 to April 2018.

### Participants and recruitment

We aimed to interview medical specialists who were *not* supportive of vaccination, working across five professional roles (paediatrician, immunologist, neonatologist, neurologist, gynaecologist) in hospitals and polyclinics, located in two cities (Yerevan, Armavir) and one province (Tavush). These sites were selected to include a mix of population size and health facility provision in different parts of the country.

For the purposes of this study, medical specialists were considered to fall into two groups:

- doctors who administer childhood vaccinations: paediatricians and immunologists in polyclinics (primary care facilities), hospital neonatologists;
- doctors who do not administer childhood vaccination but may be approached by parents to discuss vaccination: hospital paediatricians and immunologists, neurologists and gynaecologists.

Whilst paediatricians working in polyclinics would not be classified as medical specialists, their views could provide valuable insight into the first group of doctors, perhaps offering a benchmark for the quality of care we would expect.

To determine the sample size, we used the principles of “information power” [22] which consider the specificity of the study aim and the sample, the use of theory, the potential quality of the conversations and the analysis strategy.

An invitation letter was sent to the director of each health facility with an information sheet about the study, this was followed-up by a telephone call to discuss the study and secure permission to interview staff. A participant information sheet was distributed by the facility director to medical specialists who were perceived by the director to have some hesitancy about childhood vaccination. The information sheet reassured staff that taking part was entirely voluntary, and that they would not be identified. Those who were interested directly contacted the research team to discuss their participation. Medical specialists needed to consent to the interview being audio-recorded to be invited for interview. Interviews were booked with willing participants.

Recruiting medical specialists who were open about their vaccination hesitancy was a challenge. Eight doctors formally declined interviews (4 paediatricians, 1 immunologist, 1 neurologist and 2 neonatologists), giving reasons of not wishing to speak publicly about vaccinations, not wishing to be recorded, not being interested in participating or not having enough time. There were more informal refusals where nobody came forward to be interviewed, these were not documented.

### Data collection

Face-to-face interviews were conducted by staff from the NCDCP. They had a good understanding of vaccination and the health system context which was critical for a relevant in-depth discussion with appropriate probing and follow-up questions. However, they had no previous experience of qualitative research methods, so WHO research team members provided two days’ training prior to commencing the interviews and ongoing feedback on interview technique. The researchers presented themselves to participants as part of the TIP team and advised that any specific questions about vaccination or the NIP would be answered when the interview finished. Interviews were conducted in the local language at the medical specialist’s place of work. Before commencing the interview, medical specialists gave written informed consent to take part.

Interviews explored medical specialists’ role in delivering/advising on childhood vaccination (including for which vaccinations), their knowledge and views on vaccination, vaccine-preventable diseases, contraindications and AEFIs, and their skills and confidence in discussing vaccination with parents. Whilst the focus was general childhood vaccination, participants were free to discuss specific vaccinations of their choosing. An interview topic guide (see Additional File 1) was used to ensure consistency, although the format was flexible to allow participants to generate naturalistic data on what they viewed as important; as well as to acknowledge the different role of the medical specialist in vaccination. The topic guide was developed by brainstorming potential questions to explore medical specialists’ vaccination behaviours and the individual and context barriers/drivers to those behaviours (informed by the COM-B model) [15]. It was piloted with two medical specialists known to the team, after which small changes were made to some questions to improve clarity.

### Data analysis

The interviews were audio-recorded, transcribed verbatim and translated into English. The data were subjected to thematic analysis using the Framework approach [23] which is designed to address policy and programme-related questions. Analysis focused on identifying barriers and drivers to positive childhood vaccination. Similarities and differences within, and between medical specialists who administer vaccinations and those who do not administer vaccinations were investigated. The data analysis team were BS, MK, MH, SMN, CJ and KBH. Excel was used to manage the interview data.

The following steps were undertaken:

Familiarisation: CJ read five English interview transcripts (one for each professional role) to record emerging ideas and recurrent themes that were relevant to the study aims.

Constructing a thematic framework: CJ drafted the thematic framework in English; structured by the topic guide, as well as ideas and themes from the previous step. The framework was piloted by CJ and SMN using another two transcripts and refinements agreed.

Indexing and Charting: The thematic framework was then systematically applied to the interview data (by SMN and CJ). Charts were produced in Excel for each theme and summaries of responses from participants and verbatim quotes were entered. These charts were then translated into Armenian.

Mapping and Interpretation: In a 3-day workshop, the completed charts were reviewed and interrogated by the data analysis team to compare and contrast views, seek patterns, connections and explanations within the data. Team members worked from either the English or Armenian versions of the charts, and discussion was facilitated by an interpreter. Negative cases (with opposing views to the majority) were actively sought. Descriptive findings documents were jointly written for each theme in English (by SMN and CJ), then translated into Armenian for final checking.

The findings were subsequently mapped to the COM (Capability-Opportunity-Motivation) factors and their domains [13–15] by KBH.

## Results

### Participants

A total of 30 interviews were conducted with 31 participants (two participants took part in one interview) across all five professional roles (see Table 1). Most participants were paediatricians, and most were working in Yerevan. Approximately half had a remit for administering childhood vaccinations, the other half did not.

Most participants had a significant level of clinical experience, having worked in their professions for many years (paediatricians 7–43 years, immunologists 12–30 years, neurologists 10–50 years, neonatologists 5–42 years and gynaecologists 10–35 years). Only six had been in their role for 10 years or less (19%) compared to 19 (61%) with more than 25 years’ experience.

### Medical specialists’ views

Participants’ views on their childhood vaccination practices and their perceived barriers/drivers to positive vaccination practices are described here; organised by the COM factors [13–15]. Where there were differences according to whether doctors did or did not administer vaccinations, these are indicated, otherwise there were no differences. Illustrative quotes are presented throughout.

#### Childhood vaccination practices

Medical specialists unanimously confirmed that they are consulted by parents on childhood vaccination. Both groups of doctors described being asked for advice during routine consultations. Those who administered vaccination viewed this communication as part of their job. Most other medical specialists did not (discussed below in *Motivation*).

*It is not officially part of my responsibilities but because I am providing prenatal care, women who are nine months pregnant talk to us and trust us. Sometimes they seek information from us: “What is your opinion, doctor, should or should not I vaccinate my baby? Is it dangerous or not*?”

(ID2: Gynaecologist, hospital, Yerevan)

Nevertheless, it was clear they all engaged in discussions with parents.

#### Capability

The capability barriers and drivers to positive vaccination practices related to: medical specialists’ knowledge of vaccination, vaccines and vaccine-preventable diseases, knowledge of AEFIs, knowledge and skills in managing contraindications, and skills in communicating with parents about vaccination.

#### Knowledge of vaccination, vaccines and vaccine-preventable diseases

A basic level of knowledge about vaccination and vaccine-preventable diseases was evident across both groups of medical specialists. However, a more sophisticated understanding that might be expected of doctors, was not guaranteed. Neonatologists (who administer vaccinations to new-born babies) and gynaecologists particularly displayed poor understanding about immunity; offering that vaccination is too much stress for a new-born, and the immune system is better strengthened by contracting an infection naturally.

*It [vaccination] is a strong stress for immunity. You know for a new-born child which is just born, we put him/her under such stress*. (ID6: Neonatologist, hospital, Yerevan)

*It’s better to catch mumps than to be vaccinated. Same with chickenpox, it’s better to get infected with chickenpox at a younger age and strengthen the immune system, than to have problems later on. And I think it’s a bit wrong to suppress the immune system by doing many vaccinations*.

(ID8A: Gynaecologist, hospital, Yerevan)

Some doctors who did not vaccinate demonstrated poor knowledge about specific vaccines. For example, a neurologist was unsure whether the measles, mumps and rubella (MMR) vaccine causes autism, a gynaecologist believed the MMR vaccine had induced premature labour, and two other gynaecologists thought that pregnant women should not receive the flu vaccine. A lack of clarity about the safety of the HPV vaccine was particularly evident.

*The vaccination against human papilloma virus that is going to be introduced. Of course, we hear different opinions and I do not know which one to believe. The opinions are so different that I cannot decide whether the vaccine is timely, safe or not.* (ID21: Paediatrician, hospital, Yerevan)

#### Knowledge of AEFIs

Awareness of the protocol for AEFIs varied amongst medical specialists. Those who administered vaccinations were more aware of the protocol than those who did not vaccinate. It was reassuring that those who vaccinated generally stated that they follow this protocol.

*Of course, a protocol exists. In case of adverse reactions, we immediately report to the sanitary-epidemiological station, to inform them that we have had an adverse effect from this vaccine*.

(ID30: Paediatrician, polyclinic, Yerevan)

The types of AEFIs that participants mentioned reflected their role in vaccination. Those who administered vaccines were more likely to describe reactions that, whilst disquieting for parents, are not clinically significant. They knew how to manage these mild reactions.

*There can be post-vaccination reactions, well one- or two -day fever, poor appetite, sleep disorder. But these are transitory, they are not even complications. It’s a transitory state, which a child can get*. (ID9: Paediatrician, hospital, Yerevan)

The neurologists who did not administer vaccinations but were asked by parents to examine their children before or after vaccination talked more about serious AEFIs such as meningoencephalitis.

Overall, a lack of knowledge/incorrect knowledge about AEFIs was evident. Of particular concern, was the poor knowledge amongst neonatologists who are the first point of contact for parents regarding vaccination. Examples of their misperceptions were: Hepatitis B vaccination causes some babies to go yellow and have liver problems, inflammation following BCG (tuberculosis) vaccination is due to poor quality vaccine and the pentavalent vaccine leads to serious AEFIs.

Most participants either stated explicitly that they had never witnessed a serious AEFI or did not discuss this in the interview. Some gynaecologists mentioned that they had heard of serious AEFIs occurring, two of which reportedly resulted in death. Several paediatricians and neurologists observed that often what appears to be a serious AEFI is coincidental and caused by disease.

*But if someone is admitted to hospital with a serious problem, we examine and find out that it is not because of the vaccination, but it was coincided with some disease. Usually that’s the case*.

(ID21: Paediatrician, hospital, Yerevan)

#### Knowledge and skills in managing contraindications

Both groups of medical specialists reported that parents consult them about contraindications, and most were aware of a protocol which provides guidance on this. A minority, none of whom administered vaccines, stated that they used their own, or a colleague’s judgement when giving advice on contraindications (instead of using the protocol).

*Well, there are some brochures that Ministry provides, but because I am not vaccinating, I am not guided by that. I am giving advice based on my own beliefs.* (ID21: Paediatrician, hospital, Yerevan)

Knowledge of contraindications varied, irrespective of participants’ role in vaccination; with some participants incorrectly identifying minor acute infections such as a runny nose, coughing or sneezing as contraindications. Of further concern was evidence of doctors with a remit for vaccination choosing to postpone until a child is deemed to be in full health or even until they are older. This was not always due to poor knowledge, but rather a deliberate choice to “err on the side of caution” (see *Motivation*).

*If someone was sick yesterday, I will never vaccinate today. According to our new calendar it is allowed, but I am not vaccinating. I let them recover for a week and then vaccinate. Nothing will happen if we vaccinate 10 days later*. (ID25: Paediatrician, polyclinic, Yerevan)

Another precautionary strategy (also breaching protocol) was to request a second opinion from another specialist; neonatologists deferred to polyclinic paediatricians, whilst paediatricians deferred to immunologists.

#### Skills in communicating with parents

It was clear from the interview data that both groups of medical specialists offered vaccination advice to parents, even if they did not perceive it to be their role to do so. Paediatricians in polyclinics spoke most confidently about their advisory role, referring to their many years’ experience of talking to parents. The doctors who were reluctant to recommend vaccination typically attributed this to a lack of relevant expertise.

*I am not competent enough [to advise parents on vaccination], my competency is sufficient to know that the vaccinations should be done. That should be done by the primary healthcare provider. S/he is the one who was trained and the one who has the experience, I don’t have experience to offer my perspective of vaccination.* (ID1: Paediatrician, hospital, Yerevan)

Overall, most claimed that they recommended vaccination. Indeed, they described trying to convince parents about the benefits of the vaccines by explaining how diseases spread, outlining how vaccination has eradicated some vaccine-preventable diseases, and reassuring hesitant parents that vaccines are safe. However, there was evidence that this advice could be “selective”; influenced by the medical specialists’ own vaccine hesitancy about specific vaccines or their views on the seriousness of different vaccine-preventable diseases (both of which we know to be based on poor knowledge). For example, an immunologist did not think measles, mumps or rubella were serious enough to recommend vaccination. A neonatologist who was happy to vaccinate new-born babies, would not recommend vaccination for older children due to fears about side effects.

*I certainly recommend parents to think carefully [about having their old children vaccinated], to have their child checked to be prepared. I think it through with some reservations, I am not sure, to be honest. Children often develop severe reactions, some adverse events associated with the nervous system*. (ID18: Neonatologist, hospital, Yerevan)

Conversations with parents who declined vaccinations for their children were perceived to be challenging; with the doctors using different persuasion tactics, such as scaremongering, reminding parents that vaccinations are mandatory or providing information about the low rates of vaccine-preventable diseases .

*The example of chickenpox helps me a lot. I say “Do you see measles? No. Do you see mumps? No. diphtheria, tetanus? No. Do you see chickenpox? Yes. It is because we don’t get vaccinated against it, and we get vaccinated against the rest.” This example did convince that parent*.

(ID15: Immunologist, polyclinic, Tavush)

Parents’ objections on religious grounds were mentioned by half of the paediatricians and several immunologists, these were perceived to be impossible to overcome.

*I say, “What if your child gets sick? Let’s assume they get pneumonia; will you still not give any medication?” She says, “No”. “She says, “Well, whatever God’s will”. She will think that this was God’s will. You just cannot convince her.* (ID20: Immunologist, polyclinic, Yerevan)

### Opportunity

The opportunity barriers and drivers to positive vaccination practices related to medical specialists’ sources of information about vaccination, their colleagues’ advice to parents about vaccination, support from the Government of Armenia and demands from parents.

#### Sources of information about vaccination

The medical specialists were asked where they sought information about the safety and efficacy of vaccines. Participants in both groups, and in particular paediatricians working in polyclinics, reported using official guidance from the Ministry of Health. Other sources mentioned were “professional” publications and statistics drawing on evidence from other countries (notably Federation of Russia and the United States of America), often delivered within official training or workshops. A few mentioned learning from “vaccination specialists” or their colleagues.

While some also mentioned relying on mass media and social media, there were mixed views on the credibility of such sources. Notably, paediatricians in polyclinics (who administered vaccinations) were unanimously disparaging, dismissing them as an unreliable source of information. In contrast, others were less discerning about these sources of information.

*There may be various people on these shows, actors, or I don’t know, economists, who have absolutely no idea about anything. They form their opinion from things they have heard here and there. Therefore, I do not accept these and I will explain the same thing to the parents, that they should be using more reliable sources than the shows organized by the media*.

(ID28: Paediatrician, polyclinic, Yerevan)

Q. In general, what sources do you trust?

*Well, I read from health care websites, sometimes Facebook, people are also talking online.* (ID9: Paediatrician, hospital, Yerevan)

#### Colleagues’ advice to parents about vaccination

Participants in both groups reported knowing doctors who would not recommend vaccinations to parents, or who misattributed childhood maladies to vaccine side-effects. They were perceived to be poorly informed about vaccines.

*There is a group of family doctors who misinform parents, convince parents that vaccination can cause seizures, hmmm, immune deficiency. It happens where a physician, who is not even a paediatrician, has little clue about vaccinations, but advises against them*.

(ID9: Paediatrician, hospital, Yerevan)

“Other” neonatologists were described as encouraging parents to vaccinate only because it was their professional responsibility to do so and offering parents the opportunity to refuse the two vaccinations (BCG - tuberculosis, Hepatitis B) administered in the maternity hospital.

#### Support from the government of Armenia

Several medical specialists in both groups expressed anxiety about serious AEFIs occurring, and approximately half (a mix of doctors who do/don’t administer vaccinations) believed they would be held responsible should a serious AEFI occur, with no support from the Government of Armenia.

*You definitely know that if in Armenia they will vaccinate a child like that and suddenly something happens, the parent would not say that there was a problem with the child’s immune system, rather they will say that you are guilty. Definitely they will come after the doctor who did the vaccination and start fighting, you are in Armenia*. (ID1: Paediatrician, hospital, Yerevan)

The other half of the sample (including all the paediatricians working in polyclinics) knew if they followed the protocol then they would be protected. Regardless of their views on whether the Government would protect them in the event of an AEFI, the consensus amongst all participants was that they should be supported.

#### Demands from parents

Participants in both groups reported the types of vaccine safety issues that parents consulted them about: how vaccines are administered, new vaccines, adverse reactions, vaccinating when a child is unwell, the impact of vaccination on the immune system, the quality of imported vaccines, public versus private vaccines and what vaccines may contain e.g., heavy metals.

*Particularly, we have a parent, who graduated the faculty of pharmacology in our university; she received all vaccines except MMR. And she was saying “I will not receive the MMR vaccine from this particular batch, because it contains heavy metals. I don’t want my child to get these substances into her body”.* (ID15: Immunologist, polyclinic, Tavush)

Some paediatricians in polyclinics mentioned being asked about the link between MMR and autism, whilst neonatologists and gynaecologists were more likely to be questioned about the Hepatitis B vaccination. A few doctors who administered vaccinations observed that parents raise more queries nowadays because of information on the internet as well as health workers not explaining vaccination clearly to parents.

*Lately there have been more questions about all vaccines, in general, about all vaccines, and parents are refusing vaccination. Sure, I consider this is a result of our medical workers, hmm, not delivering highly professional work. Probably, we are not able to explain what the benefits of vaccination are. Because, parents are using internet, very often, hmm, there are quite different opinions*. (ID9: Paediatrician, hospital, Yerevan)

### Motivation

Motivation is affected by capability and opportunity. For these medical specialists, the motivation barriers and drivers to positive vaccination practices related to views on their vaccination role, general views on vaccination and vaccine safety, as well as their attitudes about specific vaccines.

#### Perceived role in vaccination

Unsurprisingly, the medical specialists who administered vaccinations were clear that this was an important role for them. Paediatricians and immunologists working in polyclinics were advising parents and administering childhood vaccinations daily. All the neonatologists examined new-born babies and administered the Hepatitis B and BCG (tuberculosis) vaccinations.

*Since this is a maternity hospital, vaccination is a number one service that we provide to new-born babies. There are very few contraindications, that refer to the intensive care department. All the children who are assessed to be healthy, and are with their moms, should be vaccinated. There are two vaccines, BCG and Hepatitis B, as you know, the Hepatitis B is done within the first 24 h, and BCG the first 48 h. This is what we are doing*. (ID4: Neonatologist, hospital, Yerevan)

In contrast, the doctors who did not administer vaccinations did not see a role for themselves in this area of healthcare. Indeed, all the neurologists were adamant they had no role or responsibility for advising on vaccination or giving injections, instead seeing their role as diagnosing illness and then allowing the immunologist to decide whether a child should be vaccinated.

*I want to say that it is not my responsibilities to give any advice for vaccination, because I am not an immunologist. I do my job, the patients’ treatment, the rest is the responsibility of an immunologist. I mean I don’t intervene*. (ID19: Neurologist, polyclinic, Yerevan)

#### General views on vaccination and vaccine safety

Most medical specialists in both groups stated they were in favour of vaccination, citing reasons of a reduction in the incidence of vaccine-preventable disease and eradication of some diseases (e.g. smallpox, polio). A small minority in both groups mentioned that the country of origin affected their confidence in vaccination; for example, vaccines produced in India and Korea were seen as poor quality whilst vaccines from Europe, were thought to be of good quality.

*Some time ago they were importing Indian vaccines, or something. There was a lot of talking back then. [The concern was] the quality, that, maybe, the vaccines are not good. It’s hard to tell. I don’t know. The main concern is that the adverse events are quite frequent after vaccination*.

(ID15: Immunologist, polyclinic, Tavush)

Of note were some extreme anti-vaccination views amongst neonatologists and gynaecologists (two professional groups identified to have poor knowledge – see *Capability*).

*[Vaccination is] a substance terribly affecting the organism, equivalent to an atomic bomb*.

(ID6: Neonatologist, hospital, Yerevan)

*Currently all diseases, including cancer is caused by suppressed immunity. We are forcibly suppressing immunity and then expecting the child to be active and physically well-developed in future. When the immunity is suppressed a lot of diseases occur. I think in the future there will not be preventive vaccination.* (ID29: Gynaecologist, hospital, Armavir)

#### Attitudes to specific vaccinations

There was clear evidence of hesitancy about particular vaccines: HPV, MMR and the vaccines given at birth (Hepatitis B, BCG-tuberculosis).

At the time of the study, HPV was the vaccine most recently introduced and evoked most concern about effectiveness and safety. At its most extreme level, a gynaecologist suggested that the purpose of this vaccine was to cause infertility and curb population growth.

*The Russian school is explaining that this is the vaccination that European countries want cunningly to start here. This vaccine is causing infertility, and this is already proved. The purpose of this vaccination is not cancer prevention but hindering demographic growth. Only time will prove this, is it really to prevent cancer or cause infertility?* (ID29: Gynaecologist, hospital, Armavir)

Doubts about the safety of HPV vaccine had led three participants (two gynaecologists and a paediatrician) to decline it for their daughters. However, these concerns were not uniformly expressed; some participants across both groups were in favour of the introduction of the HPV vaccine.

A small minority of medical specialists commented on suggestions of a causal link between MMR and autism. Approximately half of those who commented believed there was no evidence of a link. The other half were unsure.

*Now autism is associated with MMR. But I do not think that autism, I do not know. Only statistics can prove it. Statisticians should make decision and inform us*. (ID7: Neurologist, hospital, Tavush)

Finally, a few neonatologists and one immunologist expressed concern about the stress of BCG and HepB vaccination on a new-born’s immune system.

*I have always wondered about these ages that we have selected. Is this selection correct? I mean from zero to one-year olds, the small ones. Yes, it is dangerous, I know all that, but let’s set all that aside. Babies up to one year of age, let me be sincere and tell you. I have always wondered, that if the immune system isn’t that developed yet, is it worth it. This is what has always worried me the most*. (ID20: Immunologist, polyclinic, Yerevan)

#### Fear and self-protection

As described above, medical specialists’ personal concerns about some vaccines coupled with a perception of a lack of institutional support for doctors (see *Opportunity*) could impact on their motivation in several ways.

Doctors who did not vaccinate preferred not to discuss or recommend vaccination to parents; whilst some doctors who did vaccinate would refer parents to other doctors for a “second opinion” and use false contraindications to postpone or avoid vaccination. This seemed to be related to self-protection and a fear of being blamed.

*We don’t have this kind of support in any sphere. We are the most vulnerable professional group of this country.*

Q. Does this kind of situation affect your readiness to advise parents to vaccinate their kids?

*Yes, yes, of course it affects me.* (ID10: Gynaecologist, hospital, Armavir)

## Discussion

This is the first in-depth qualitative study in Armenia to explore the vaccination views and experiences of medical specialists who do not have an official remit for administering vaccination; and to compare these with doctors who are responsible for vaccination. It uncovered significant, complex and inter-related capability, opportunity and motivation barriers to positive childhood vaccination practices. These could potentially threaten the ongoing success of the Armenian childhood vaccination programme. Our findings contribute to the emerging evidence base on health worker vaccination behaviours in Central and Eastern Europe [9–11] and to the portfolio of TIP projects focused on health workers [24, 25]. It addresses a gap in the global literature to highlight the important role that these groups of medical specialists have in the promotion of childhood vaccination and the support they need to do this better.

The take home finding was that medical specialists in both groups were routinely approached by parents to advise on vaccination across a wide range of topics; and they all engaged in these conversations, even those who believed it was not their job and they did not have the expertise to do so. The consensus was that they recommended vaccination, but those without a remit for vaccination were selective in these recommendations reflecting their own vaccine hesitancy. A “committed, confident and competent vaccination workforce” is vital to achieving high vaccination coverage [26, p2601]. If this wider group of specialist doctors are to be part of the vaccination workforce in Armenia, they have a duty to do this well. This responsibility is intensified because, in Armenia, medical specialists are held in higher esteem by patients than primary care doctors (personal communication within research team). Our study findings revealed there is considerable room for improvement.

Whilst the study was focused on childhood vaccination generally, a second striking finding related to the HPV vaccine. Both groups of doctors appeared less knowledgeable and less confident in its safety. We heard mixed views about its introduction with a small minority having already declined it for their own daughters. These interviews coincided with the early roll out of this vaccination, when there was considerable controversy surrounding the vaccination [20, 26].

We interviewed some polyclinic paediatricians who have primary responsibility for the childhood vaccination programme. Our expectation was that they would be committed, confident and competent [27]. Primary care doctors are an influential source of vaccination information for parents; and parents want them to be receptive to, and skilled in, discussing their concerns [16]. It was reassuring that these doctors were generally well informed (except about AEFIs and HPV), mainly positive about vaccination and guided by official information and protocols. They felt comfortable in their advisory role, apart from conversations with parents declining vaccination for their children which is a challenge not unique to Armenia [24, 25, 28].

A concern was their use of false contraindications and seeking a second opinion to postpone or avoid vaccination, confirming previous reports [7, 8, 10]. This was occurring even though they believed that they would be supported should there be a serious AEFI. The risk is that this leads to missed opportunities for vaccinating children and can indirectly contribute to vaccine hesitancy among parents because it creates a false safety concern [28]. Again, the problem is not unique to Armenia [24, 25, 29, 30]. Elsewhere, these practices have been linked to the absence of clear guidelines [29, 30]. However, in Armenia protocols are in place, and our data suggest these behaviours are prompted by fear of repercussions should an adverse event occur. These primary care doctors are required to attend annual immunization training updates. Based on the findings, our recommendation would be that these sessions should include updates on AEFIs and HPV, training on vaccination communication skills [31, 32] (supported by a new guideline for communication) as well briefing on the legal position of doctors who are recommending and administering vaccinations. We also recommend that the rolling programme of training for primary care doctors that has been implemented due to the COVID-19 pandemic, is used as another opportunity to address these knowledge and skill gaps.

The other doctors interviewed with a remit for childhood vaccination were hospital neonatologists. Here the findings were less positive. We heard evidence of poor understanding about the immune system and AEFIs, some negative vaccination attitudes and practices. One neonatologist shared some concerning personal anti-vax views. We could not find any studies to compare these findings with. However, we know that failure to receive the birth vaccines is a risk factor for incomplete vaccination up to two years of age [33, 34] even in a country, like Armenia, where childhood vaccination is mandatory [33]. Refusal of birth vaccines may be the first indication that parents are vaccine hesitant, meaning that neonatologists have a crucial role to play in recommending vaccination and addressing parents’ concerns. In Armenia, these doctors are not required to attend the annual vaccination training updates. Based on these findings, our recommendation is that they should be.

The assumption at the outset of this study was that there were medical specialists who do not have a remit to vaccinate and were recommending *against* vaccination. This was difficult to confirm. Armenian research team members believe our participants may have been more positive in their interviews than they truly felt. That said, we heard enough to be concerned. Amongst these doctors there was evidence of poor knowledge, vaccine hesitancy, anti-vax beliefs, disregard of vaccination protocols, misplaced fear of being blamed and unsupported for AEFIs, over reliance of media and social media sources and a clear reluctance to have a role in vaccination. Yet these doctors continued to discuss vaccination with their patients. As with the neonatologists we found no childhood vaccination studies with which to compare our findings. Gynaecologists are reported elsewhere to be less positive than family physicians in their HPV vaccination recommendations [20] and reluctant to discuss flu vaccination with pregnant women [21]. Key drivers to health workers recommending vaccines are knowledge of vaccines and vaccination, confidence in their efficacy and safety, awareness of policies, and perceptions of social endorsement and support from colleagues [20, 35]. Our recommended interventions were: integrate technical vaccination education and communication skills into medical school training and existing CPD courses for these different specialists; to develop brief, focused protocols specific to their role, and identify trusted role models within their speciality to promote vaccination. Indeed, since the end of the TIP project, specific training on immunization has been introduced into several medical schools, and one-day trainings have been implemented for hospital-based medical specialists. There is further work to do.

Finally, it is important to reflect on the strengths and limitations of this study. First, we heard from medical specialists who did not have a remit to administer vaccinations but were involved in conversations about vaccination with parents. The perspectives of these groups of health workers are missing in the literature yet are important, particularly in countries where referring to, or independently seeking advice from, such experts is a widely used practice. In comparing them with doctors who did administer vaccinations, across hospital and polyclinic settings, we could uncover important differences in their barriers to positive vaccination practices to develop tailored strategies. In reflecting on whether we achieved generalizability (as a qualitative concept) [22], we recruited participants across professions, regions and primary/secondary care where over three quarters had over ten years of clinical experience, reflecting the profile of Armenian doctors. We achieved data saturation (where no new themes were emerging) and captured good diversity of views and practices, providing a valuable breadth of insight. We, therefore, have no reason to think that our participants’ accounts would be different to other medical specialists in different health facilities or locations. That said, it was difficult to recruit medical specialists who were willing to speak openly against childhood vaccination. Eight doctors declined, and others did not volunteer to participate. This means that our findings may be more positive than the true situation at that time, and highlights the importance of urgent action, perhaps most urgently with neonatologists. Since the study was conducted, the 2018 Armenian Revolution has happened, and it is the belief of our Armenian authors that doctors today would speak more openly. It would be interesting to repeat this study now. Furthermore, time and resources prevented us from interviewing some parents to understand their reasons for approaching these medical specialists about childhood vaccination. Future research could usefully include their important perspective.

Second, the TIP approach [13, 14] and use of the COM-B model [15] during data analysis provided a comprehensive, theory-informed approach to identify determinants of behaviour.

A final strength of this work was that we built qualitative research capacity in Armenia by training staff from the NIP to conduct all steps of the study. An added benefit was drawing on their existing topic and context knowledge to interpret the findings.

## Conclusion

Applying a structured, comprehensive and theory-informed approach with stakeholder engagement and active listening to a critical vaccination influencer group allowed us to identify critical issues related to childhood immunization in Armenia, as well as possible opportunities to respond to these. The study revealed that general high vaccination coverage may disguise underlying issues such as those that relate to false contraindications and postponement of vaccination. The study also addressed important gaps in the literature as it focused on a geographical sub-region which is almost absent from the literature on vaccination uptake and hesitancy, and related to a practice that is widely used in this sub-region, namely the role of medical experts of various kinds in advising parents about vaccination.

## Electronic supplementary material

Below is the link to the electronic supplementary material.

Supplementary Material 1

## Data Availability

The datasets generated during and/or analysed during the current study are not publicly available due to participants not consenting to this and our concerns about deductive disclosure (there are small numbers of medical experts in these health facilities), but are available from the corresponding author on reasonable request.

## List of abbreviations

AEFI: Adverse events following immunization
COM-B: Capability-Opportunity-Motivation-Behaviour model
HPV: Human papillomavirus
MMR: Measles-mumps-rubella
NCDCP: National Centre for Disease Control and Prevention
NIP: National Immunization Programme
TIP: Tailoring Immunization Programmes
WHO: World Health Organization
