# Supplementary material for: Medical specialists’ attitudes and practices towards childhood vaccination: a qualitative study in Armenia
Source: BMC Pediatr. 2022 Oct 29;22:620. doi: 10.1186/s12887-022-03687-3 (PMC9617035; doi:10.1186/s12887-022-03687-3)
Supplement: Supplementary file 1 — Supplementary Material 1 [file 12887_2022_3687_MOESM1_ESM.docx]

**INTERVIEW TOPIC GUIDE**

**Understanding medical experts’ attitudes and behaviours towards childhood vaccination in Armenia**

| **1. INTRODUCTORY QUESTIONS** |
| --- |
| - What do you do on a daily basis? - How long have you been in this role? |

| **2. BEHAVIOURS** |
| --- |
| **2a. What is your role in administering or advising on vaccinations?**   - Is that an official part of your job? If no, how did you become involved in this?   *If they administer vaccinations*   - Which vaccinations? - Children up to what age? - Teenagers? - Catch-up vaccinations? - Adults? - How often are you providing these vaccinations?   *If they advise on vaccinations*   - Which vaccinations? - Who are you advising? - How often are you advising about vaccinations? When does this occur?   *If neither*   - Can you see a time where you would be asked to discuss vaccination with your patients? Please tell me about that. **GO TO SECTION 2d.** |
| **2b. What advice on childhood vaccination do you give?**   - Under what circumstances do parents come to you about vaccination (are they referred to you; do parents take the initiative; do you take the initiative)? - Why that advice? - What information do you use to form your advice? What information do you trust? - What are the issues that parents raise? How do you address this? - Does your personal view influence your conversations with parents about childhood vaccination?   - If yes, how? - What advice do you give to friends and family about immunisation?   - Is this the same or different as you give to your patients? |
| **2c. How confident are you in talking to parents about vaccination? Why is that?**   - What about talking to hesitant parents? - Parents who are anti-vaccination? - Parents from religious communities? - Are there any other groups that you consider to be more hesitant to vaccination? Who? Why do you think this? |
| **2d. What do you think are contraindications to childhood vaccination?**   - Is there a protocol? Have you seen it? Do you follow it? - What do you advise parents about this? - Do some parents ask you to diagnose (false) contraindications to vaccination as an easy way to avoid vaccination? If yes, what do you do? - How confident are you in discussing contraindications with parents? - How often are you unsure? What do you do when you are unsure? Why is that? |
| **2e. What do you know about AEFIs (adverse events following immunisation)?**   - Is there a protocol? Have you seen it? Do you follow it? - What do you advise parents about this? - How confident are you in discussing these with parents? - How often are you unsure? What do you do when you are unsure? Why is that? - What do you think would happen in case of an AEFI following vaccination where the parents believe the event was caused by vaccination?   - Would you be held responsible if you had advised them to vaccinate their child?   - Would anyone support or protect you?   - *If not -* how would you like to be supported? At what level should the support be? (health care facility, region, national) Why that level?   - Does this situation affect your willingness to promote childhood vaccination with parents? How? - What would happen if the opposite occurred – a health worker advised against vaccination and then the children caught the disease? |

| **3. ATTITUDES** |
| --- |
| **3a. What are your own attitudes towards vaccine-preventable diseases (VPDs) and childhood vaccination?**  ***(Note. we may want to probe particular VPDs and vaccines in these questions)***   - What are the risks to local children of VPDs? Any VPDs that you are particularly concerned about? - How serious would the VPDs be for local children? - Are there any risks of vaccines to local children? - How do you weigh up the risks and benefits of vaccines and VPDs? - What information have you used to form your opinion? Why those sources? How much do you attend to the messages in the media? |
| **3b. What are the attitudes of your colleagues?** |

| **4. IDEAS FOR SUPPORTING MEDICAL EXPERTS TO PROMOTE CHILDHOOD VACCINATION** |
| --- |
| **4a. What can the Ministry of Health do to support medical experts like yourself to promote childhood vaccination?**  (*Some ideas if they don’t have any)*: training in vaccination, training in communication skills, other medical experts (like them) championing vaccination, published evidence  REMEMBER TO ASK ABOUT ANY CHALLENGES THEY IDENTIFIED IN THE INTERVIEW   - Is there anyone else could provide this support? - Which of these strategies would make the most difference to you?   **4b. What can the Ministry of Health do to change the view of medical experts who are anti-vaccination?** |

| **5. FINAL QUESTION** |
| --- |
| **Is there anything else about vaccination that you want to tell me before we finish?**   - Anything that we haven’t discussed? |

**END INTERVIEW**
